# Hand dexterity, not handgrip strength, is associated with executive function in Japanese community-dwelling older adults: a cross-sectional study

**DOI:** 10.1186/s12877-018-0880-6

**Published:** 2018-08-24

**Authors:** Kimi Estela Kobayashi-Cuya, Ryota Sakurai, Naoko Sakuma, Hiroyuki Suzuki, Masashi Yasunaga, Susumu Ogawa, Toru Takebayashi, Yoshinori Fujiwara

**Affiliations:** 1 Research Team for Social Participation and Community Health, Tokyo Metropolitan Institute of Gerontology, 35-2 Sakae-cho, Itabashi-ku, Tokyo 173-0015 Japan; 2 Department of Preventive Medicine and Public Health, School of Medicine, Keio University, 35 Shinanomachi, Shinjuku-ku, Tokyo 160-8582 Japan

**Keywords:** Cognitive function, Community-dwelling older adults, Executive function, Hand dexterity, Handgrip strength

## Abstract

**Background:**

An association between handgrip strength, hand dexterity and global cognition is suggested; however, it is unclear whether both hand motor functions are associated with executive function, which is important for performing daily activities. Understanding this association will help identify motor risk factors for impairment of executive function in late adulthood. We aim to investigate the relationship of handgrip strength and hand dexterity with executive function in physically and mentally healthy community-dwelling older adults.

**Methods:**

Three hundred and twenty-six older adults (287 women, mean age ± SD, 70.1 ± 5.6) underwent handgrip strength and hand dexterity tests using a hand dynamometer and the Purdue Pegboard Test (PPT), respectively. Executive function was evaluated with the Trail Making Test (TMT)-A, TMT-B and Digit symbol; global cognition was assessed with the Mini-Mental State Examination (MMSE).

**Results:**

Age-group differences showed that the younger groups (60–64, 65–69 and 70–74) had a significant better PPT and executive function performance than the oldest group (75 and older), whereas no significant age differences were observed for handgrip strength. Multiple regression analysis adjusted for potential covariates, including MMSE scores, showed that TMT-A, TMT-B, and Digit symbol were significantly associated with PPT scores; however, no significant association was observed between executive function variables and handgrip strength.

**Conclusions:**

Hand dexterity is vulnerable to the effects of aging and, contrary to handgrip strength, it strongly associates with executive function, independent of global cognition. Our results suggest that assessing hand dexterity may help identify individuals at higher risk of impairment of executive function among high-functioning older adults.

## Background

Cognitive decline is a major clinical and public health concern that threatens the quality of life of older adults and their families and poses significant challenges to aging societies [1]. Several behavioral studies have been conducted to understand the risk factors for cognitive decline and the incidence of dementia and found that impairment of motor function is closely related to these changes [2–4]. A better understanding of the association between motor and cognitive performance in older adults without cognitive impairment could help accurately detect at early-stage which individuals have motor risk factors associated with cognitive decline.

Cross-sectional studies have indicated that either handgrip strength [5, 6] or hand dexterity [7] is associated with global cognitive performance. Also, clinical studies have reported significant differences in motor impairment (loss in muscle control or movement) between cognitively normal older adults and those with mild cognitive impairment (MCI) [3, 8]. These findings are reasonable because handgrip strength and coordination are needed to successfully perform daily activities that require cognitive engagement such as writing, cooking, gardening, making craft-works, and playing instruments. The nature of these hand movements requires visual search [9], motor speed skills [10], attention allocation and motor planning [11]. Therefore, executive function, which consists of cognitive processes of attention, working memory, planning, judgment, task flexibility, and inhibition [12], seems to be strongly influenced by the level of hand motor function.

Although there is a growing body of epidemiological evidence for the association between hand motor function and cognitive function [13], it is still unclear which hand motor variable including handgrip strength and hand dexterity is strongly associated with executive function. Executive function is a cognitive domain that is important for maintaining a safe and independent living in older adults [12]; however, reduced executive function seems to be prevalent even among healthy, community-dwelling older adults without overt cognitive impairment [4]. For this reason, it is crucial to evaluate the association between hand motor function and cognitive performance in cognitively intact subjects in order to provide more evidence of the motor risk factors associated with cognitive decline in late adulthood.

Therefore, our aim is to understand which hand motor variable (focusing on handgrip strength and hand dexterity of the dominant hand) is strongly associated with executive function in healthy community-dwelling older adults aged 60 years and over with intact global cognition. We also examined whether significant variability exists in hand motor and executive function variables across age groups in our sample. As mentioned earlier, executive function is a significant predictor of functional status and independent living determined by ADLs (activities of daily living) and IADLs (instrumental ADLs) [14]; thus, understanding its association with hand motor function would contribute to identify older adults at higher risk of impairment of executive function.

## Methods

### Study design and population

The sample consisted of 326 physically and mentally healthy Japanese community-dwelling older adults recruited from our volunteer database available from a longitudinal and randomized controlled trial of the REsearch of PRoductivity by INTergenerational Sympathy (REPRINTS) program at Tokyo Metropolitan Institute of Gerontology (TMIG) between 2004 and 2017. Participants were recruited from three types of areas—urban (Bunkyo, Chuo, Itabashi and Toshima Wards, Tokyo), suburban (Kawasaki city, Kanagawa), and rural (Nagahama city, Shiga)—through community newsletters and meetings. The REPRINTS program is a longitudinal study, with no significant effects on hand motor function, which evaluates the effects of an intergenerational book-reading program on cognitive function in community-dwelling older adults aged 65 years and older [15]. A randomized, short-term study of this program includes middle-aged adults and over. In the present study, the analyzed data was combined from the two databases, so the number of cases for one variable (Digit symbol, see below) was 207.

The exclusion criteria included history of cerebrovascular disorder, history of hospitalization due to an acute medical condition (e.g., stroke and heart disease) within 3 months before the study; motor/neuromuscular problems (e.g., hand tremors); significant hearing loss and visual deficits; and mental disorders and cognitive impairment indicated with a Mini-Mental State Examination (MMSE) score of 25 or lower [16]. Handedness was determined according to the Edinburgh Handedness Inventory [17]. In this regard, to examine the motor function of the dominant hand, participants with ambidexterity were excluded. However, only the data of the dominant right hand is reported due to the lack of left-handed participants (see Results). Written informed consent was obtained from all participants before examination. The study was conducted in accordance with the Declaration of Helsinki (1983). The Ethics Committee of the Tokyo Metropolitan Institute of Gerontology approved the research protocol.

### Hand motor tests

#### The Purdue Pegboard Test (PPT)

The PPT (Lafayette Instrument Company, Model 32,020) is a hand and finger dexterity test [18]. It consists of a 19.7 × 44.9 cm board with 25 slotted holes in a 5 × 5 array. The participants were instructed to insert one pin at a time starting from the top hole in either the right or left row, depending on the starting hand, as fast as possible for 30 s without option to pick up any dropped pins. The order of the starting hand was counterbalanced across participants. There was one practice session for each hand in which participants could practice until they were able to insert five pins in a row. The number of pins correctly inserted in 30 s was recorded for each trial, and the average of two trials was used for analysis.

#### Handgrip strength

Handgrip strength was measured on the dominant hand using the Smedley dynamometer (ES-100, Evernew Co., Ltd., Koto., Tokyo, Japan) to the nearest 0.5 kg of force. Subjects were instructed to deeply inhale and fully exhale while squeezing the dynamometer with as much force as possible on their dominant hand. The average of two trials was used for analysis.

### Neuropsychological tests for measuring executive function

#### The Trail Making Test (TMT)

The TMT has been widely used for assessing executive function and involves cognitive skills including visual search, perceptual/motor skills, processing speed, attention, switching, and working memory [19, 20]. It consists of two parts: The TMT-A, which requires the subject to draw a line connecting consecutive encircled numbers (1 to 25) randomly distributed in a sheet form, and the TMT-B, which requires the subject to alternate between numbers (1 to 13) and letters of Hiragana, a Japanese syllabary, (i.e., 1-A) as quickly and as accurate as possible. The TMT-A has been associated with cognitive skills involved in executive function such as visual search, processing speed, sustained attention and working memory [19–22], while the TMT-B requires additional executive function skills such as task switching, cognitive flexibility and greater working memory [23]. The time (in seconds) required for the participant to complete each task was used for analysis.

#### Digit symbol - Wechsler Adult Intelligence Test-Revised (WAIS-R)

The Digit symbol subtest of the WAIS-R consists of a row of squares containing symbols paired with digits from 1 to 9 and another row of blank squares randomly assigned with digits from 1 to 9. After a practice session, the participant fills in the blank squares with the symbol that corresponds to each digit. The Digit symbol test has been used to evaluate executive function [24], where sustained attention, visuomotor coordination, and response speed contribute to the performance of the test [25]. The total score was the number of squares correctly matched in 90 s.

### Covariates

All participants were interviewed by a physician to assess heath related characteristics that could be associated with hand dexterity and executive function including demographics such as age, sex and years of education. Functional health status was evaluated using the Tokyo Metropolitan Institute of Gerontology Index of Competence (TMIG-IC), in which scores ranging from 0 to 13 indicate functional capacity in IADLs (i.e., being able to shop, prepare meals), intellectual activities (i.e., reading newspapers, books) and social roles (i.e., visiting friends) [26]. Comorbidities consisted of the presence or absence of self-reported diseases including heart disease, diabetes, hypertension, and stroke. Depressive mood was assessed using a 15-item short version of the Geriatric Depression Scale (GDS-15), a measurement of depressive feelings during the past week. Higher GDS scores indicate greater depression. Scores of five and above are indicative of depressive symptoms [27, 28]. Global cognition was evaluated using the MMSE, a cognitive screening test both for the evaluation of general or global cognitive performance and for detecting dementia [29]. The test consists of 30 items of various domains including orientation, attention, immediate recall, delayed recall, language, and visuospatial ability. It has been shown that the total scores of the MMSE and the Montreal Cognitive Assessment (MoCA), a brief cognitive screening test with higher sensitivity to detect MCI than the MMSE [30], are significantly correlated even among participants who score > 24 [29]. We have therefore included MMSE as a covariate to adjust for global cognitive function despite their restricted range of score in the present study.

### Statistical analysis

All data were analyzed using the Statistical Package for the Social Sciences (SPSS) 23.0 (SPSS Inc., Chicago, Ill., USA). Simple correlations were conducted among hand motor function and cognitive variables. To examine the differences in hand motor and cognitive variables among four age-groups, a one-way ANOVA was performed. A Bonferroni correction for *p* <  0.008 was applied in post-hoc comparisons (0.05/6) to avoid type 1 error, where the denominator 6 represents the six pairwise comparisons resulting after comparing the four age-groups. Multiple regression analyses controlling for age, sex, years of education, TMIG-index of competence, depression, comorbidities, and MMSE tested the associations between executive function (i.e., TMT-A, TMT-B and Digit Symbol as dependent variables) and hand motor function (i.e., handgrip strength and hand dexterity as independent variables). Regression analyses were performed separately for each independent and dependent variable (Model 1). In total, six regressions were run in Model 1. Also, to eliminate the confounding variable between hand motor variables, regression models including both handgrip strength and hand dexterity as independent variables together were then performed separately for each dependent variable (Model 2). Thus, three regressions were run in Model 2. Statistical significance was set at *p* <  0.05.

## Results

Five participants who showed significant cognitive decline impairment (MMSE < 26) and 12 participants who were ambidextrous were excluded from the analyses. Thus, a total of 326 right-handed older adults were included in the analyses. Table 1 shows age-group differences in hand motor variables, cognitive variables and covariates. After dividing the sample into four age-groups, performance in PPT, TMT-A, and Digit symbol were significantly higher in the younger groups (ages 60–64, 65–69, and 70–74) than the oldest group (≥75), whereas no significant age-group differences were observed in handgrip strength. TMT-B scores were also significantly different between age-groups 60–64, 65–69 and the older groups, whereas MMSE scores were significantly different between 60 and 64 and ≥ 75. Years of education was significantly different between the youngest group (60–64) and the older groups (70–74 and ≥ 75). The proportion of participants with heart disease and hypertension tended to be higher in older groups.

**Table 1:** Comparison of covariates, hand and cognitive variables among age categories (*N* = 326)

Variables	Age categories				*p*-value
	60–64 (*n* = 54)	65–69 (*n* = 104)	70–74 (*n* = 101)	≥ 75 (*n* = 67)	
Years of education	14.4 ± 2.5	13.4 ± 2.2	13.3 ± 2.5^⁎^	12.8 ± 2.7^⁎^	0.004
TMIG-IC	12.2 ± 0.8	12.3 ± 0.9	12.4 ± 0.9	12.2 ± 1.3	0.645
GDS	2.3 ± 2.1	3.1 ± 2.5	2.5 ± 2.3	3.0 ± 2.3	0.09
Heart disease, n (%)	2 (3.7)	1 (1.0)	11 (10.9)	7 (10.4)	0.012^a^
Diabetes, n (%)	2 (3.7)	9 (8.7)	6 (5.9)	5 (7.5)	0.670^a^
Hypertension, n (%)	11 (20.4)	23 (22.1)	27 (26.7)	28 (41.8)	0.020^a^
Stroke, n (%)	3 (5.6)	6 (5.8)	5 (5.0)	3 (4.5)	0.983^a^
Hand motor variables, mean ± SD					
Handgrip strength (Kg)	23.4 ± 4.7	22.6 ± 5.3	22.9 ± 7.0	20.9 ± 6.3	0.104
PPT (Number of pegs)	14.5 ± 1.5	13.7 ± 2.1	13.1 ± 1.9^⁎^	11.9 ± 1.9^⁎,†,‡^	< 0.001
Cognitive variables, mean ± SD					
MMSE	29.2 ± 0.9	28.8 ± 1.3	28.9 ± 1.0	28.6 ± 1.3^⁎^	0.023
TMT-A	30.2 ± 7.7	35.3 ± 13.2	37.9 ± 11.1^⁎^	43.2 ± 12.4^⁎,†,‡^	< 0.001
TMT-B	74.5 ± 18.6	91.9 ± 37.6^⁎^	107.4 ± 39.9^⁎,†^	121.6 ± 40.8^⁎,†^	< 0.001
Digit symbol^b^	67.7 ± 9.3	60.2 ± 13.8	55.3 ± 10.9^⁎^	45.6 ± 8.4^⁎,†,‡^	< 0.001

Values are expressed as mean ± SD

*TMIG-IC* Tokyo Metropolitan Institute of Gerontology - Index of Competence, *GDS* Geriatric Depression Scale, *MMSE* Mini-Mental State Examination, *TMT* Trail Making Test

Bonferroni correction for post-hoc tests: ^*^p <  0.008 vs. 60–64; ^†^*p* <  0.008 vs. 65–69; ^‡^*p* < 0.008 vs. 70–74

^a^The Chi-square test was performed

^b^The total number of subjects analyzed was 207

**Fig. 1:**
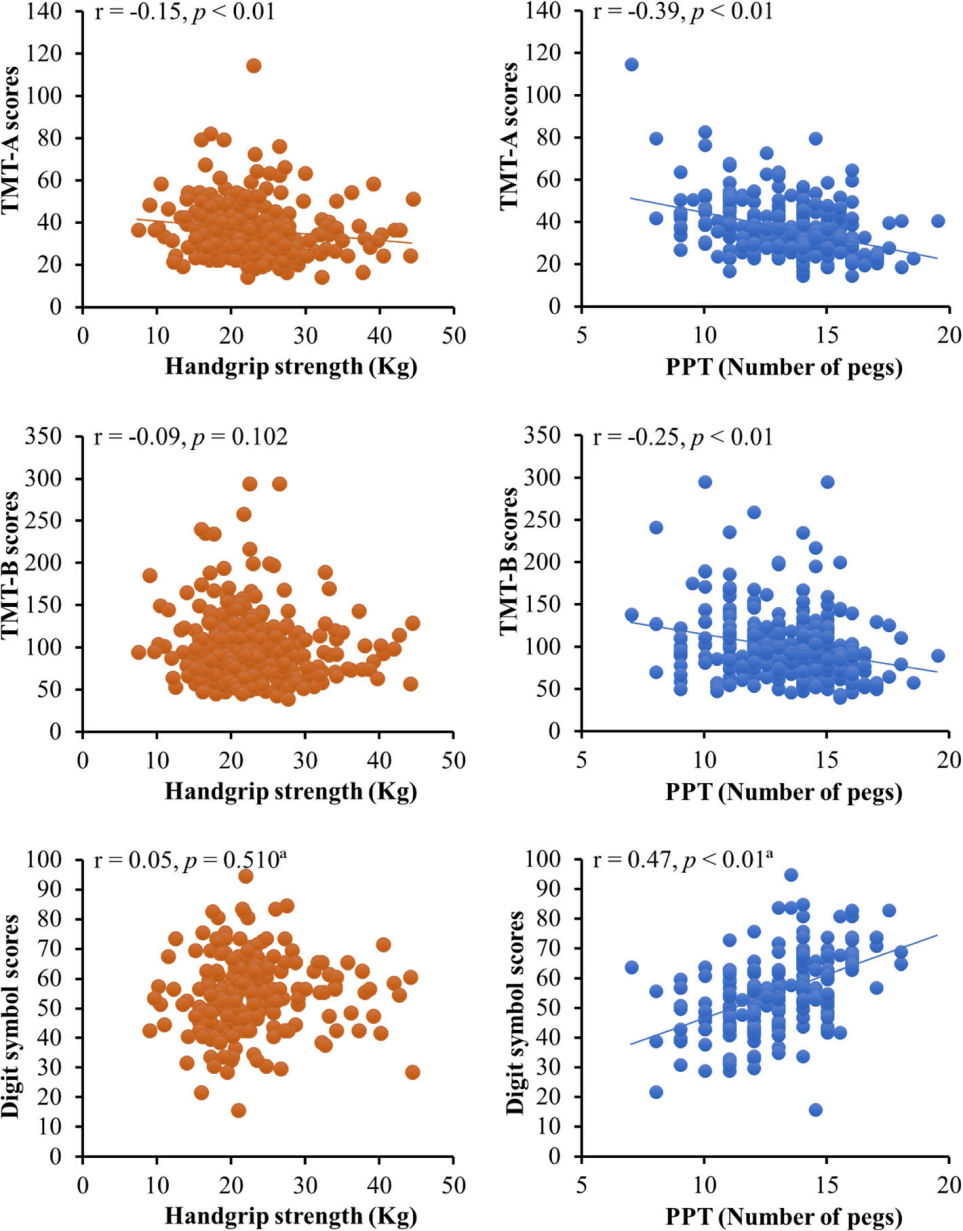
Scatter diagrams of the relationships between hand motor variables (handgrip strength, PPT performance) and executive function variables (TMT-A, TMT-B, Digit symbol). Each plot shows the best-fit simple regression line, the correlation coefficient (*r*) and the statistical significance (*p*). ^a^The total number of subjects analyzed was 207

**Fig. 2:**
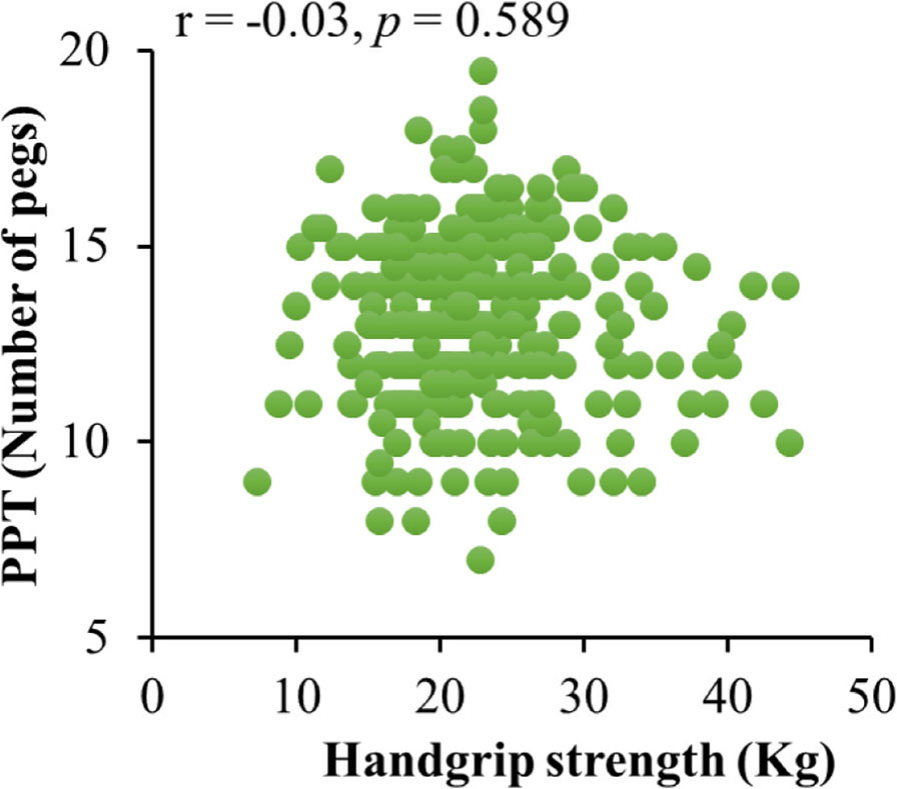
Scatter diagram of the relationship between handgrip strength and hand dexterity, measured by PPT performance. The plot shows the best-fit simple regression line, the correlation coefficient (*r*) and the statistical significance (*p*)

Figure 1 shows the scatter plots of the associations between executive function variables (TMT-A, TMT-B, Digit symbol) and hand motor variables (handgrip strength and PPT performance) in older adults. Correlation analyses showed that PPT performance, which was assessed by the number of inserted pegs, was significantly correlated with TMT-A, TMT-B and Digit symbol. Contrarily, handgrip strength was not significantly correlated with TMT-B and Digit symbol; only TMT-A showed a significant but low correlation coefficient with handgrip strength. On the other hand, Fig. 2 shows no significant association between handgrip strength and PPT performance.

Table 2 shows the results of the multiple regression analyses evaluating the association between executive function (i.e., TMT-A, TMT-B and Digit symbol) and hand motor function (i.e., handgrip strength and hand dexterity). Lower PPT performance was associated with longer both TMT-A and TMT-B times and lower Digit symbol score. These associations remained significant after adjusting for potential covariates and additional adjustment for handgrip strength. On the other hand, no association was observed between executive function variables and handgrip strength, even after additional adjustment for PPT scores. Covariates including age (*p* <  0.001), sex (*p* = 0.016), GDS (*p* = 0.025), stroke (*p* = 0.039) and MMSE scores (*p* = 0.005) significantly contributed to the association between TMTA and PPT performance, whereas only age (*p* <  0.001) and MMSE scores (*p* = 0.13) contributed to the association between TMTB and PPT performance. Age (*p* <  0.001), stroke (*p* <  0.001) and diabetes (*p* = 0.013) significantly contributed to the association between digit symbol and PPT performance.

**Table 2:** Multiple Linear Regression Model Summary for TMT-A, TMT-B and Digit symbol

Dependent variables	Independent variables							
	Model 1				Model 2			
	HG		PPT		HG		PPT	
	β (95% CI)	*p*-value	β (95% CI)	*p*-value	β (95% CI)	*p*-value	β (95% CI)	*p*-value
TMT-A	−0.12 (− 0.55, 0.06)	0.114	−0.33 (−2.60, − 1.32)	< 0.001	−0.33 (− 2.58, − 1.29)	< 0.001	−0.09 (− 0.47, 0.10)	0.177
TMT-B	−0.02 (−1.11, 0.84)	0.782	− 0.12 (− 4.38, − 0.06)	0.044	−0.12 (− 4.38, − 0.04)	0.046	−0.01 (− 1.04, 0.90)	0.888
Digit symbol^a^	0.02 (− 0.39, 0.31)	0.827	0.30 (1.08, 2.56)	< 0.001	0.30 (1.08, 2.56)	< 0.001	0.03 (−0.39, 0.28)	0.751

Each of the regressions was performed separately for each independent variable (TMT-A, TMT-B, and digit symbol)

Model 1: includes two hand variables analyzed separately as independent variables and adjusted for age (a continuous variable), sex, years of education, TMIG-Index of Competence, GDS, hypertension, stroke, heart disease, diabetes and MMSE

Model 2: includes two hand variables analyzed together as independent variables and adjusted for the same covariates as Model 1

*CI* Confidence Interval, *HG* Handgrip strength, *PPT* Purdue Pegboard Test

^a^The total number of subjects analyzed was 207

## Discussion

The objective of this study was to evaluate which hand motor variable (handgrip strength or hand dexterity) has a stronger association with executive function performance in community-dwelling older adults. Our results revealed that hand dexterity assessed by PPT performance, and not handgrip strength, was significantly associated with executive function among high-functioning older adults. Regression analysis confirmed this association after further adjustment for covariates including handgrip strength. These findings suggest that hand dexterity may be considered a measurable motor risk factor for the early detection of executive function impairment among older adults with intact global cognitive performance. To our knowledge, the present study provides the first evidence that hand dexterity is more strongly associated with executive function performance than handgrip strength among physically and mentally healthy community-dwelling older adults.

The rationale of this association resides in that hand dexterity requires not only the sensorimotor coordination of hands and fingers with the eyes [31], but it also requires complex cognitive processes observed in executive function such as attention, working memory, planning, judgment, task flexibility, and inhibition [12]. These cognitive processes seem to play a significant role in the successful performance of fine motor movements rather than that of muscle strength. This finding may also be attributed to the commonality of the association between the prefrontal cortex of the brain with executive function [32] and hand dexterity [33], as well as the association between nerve myelination with executive function [34] and hand dexterity [35]. All these shared complex mechanisms make hand dexterity more closely related to executive function impairment than handgrip strength.

The association between the hand motor variables (handgrip strength and hand dexterity) was also evaluated since they are essential hand components for performing manual activities. In this regard, a previous study reported an association between handgrip strength and hand dexterity in a sample with a wide age range (ages 18 to 93) [36]; however, in the present study with community-dwelling older adults, handgrip strength was not significantly associated with hand dexterity. A possible explanation is that our participants were high-functioning individuals with no significant differences in handgrip strength. Although reduction in muscle mass is associated with reduced handgrip strength, possibly influencing hand dexterity performance in older adults, the significant association between hand dexterity and executive function found in the present study suggests that the integration of complex cognitive and sensory mechanisms constitutes a crucial component of hand motor function. This may be further supported by the age-group differences in executive function and hand dexterity performance observed in the present study, suggesting hand dexterity as a useful motor variable to evaluate executive function in different age subgroups of older adults.

Executive function has been strongly associated with independent living [14], and it is considered a stronger predictor of functional impairment than MMSE in healthy subjects and in patients with MCI and Alzheimer’s disease [12, 37]. Therefore, the association observed in the present study between executive function and hand dexterity also suggests the role of hand dexterity in maintaining executive function in high-functioning older adults.

The strength of this study includes the analysis of hand motor and cognitive variables in a large sample of active and cognitive intact community-dwelling older adults. This study also provides an understanding of the importance of hand dexterity as a measurable hand motor indicator for the early detection of impairment of executive function in high-functioning older adults. However, the findings of the current study need to be interpreted with caution. First, this is a cross-sectional study, and therefore we cannot establish a cause and effect relationship between hand dexterity and executive function; longitudinal studies are needed to elucidate this association. Second, only TMT and Digit symbol tests were used to evaluate executive function; therefore, more executive function tests should be used to provide further support to the association with hand dexterity. Third, the PPT evaluates finger and hand dexterity; however, due to the complexity of fine motor function, it is necessary to include more hand dexterity tests to better understand the association with executive function.

## Conclusions

The present study showed that hand dexterity, not handgrip strength, associates with executive function variables in community-dwelling older adults with intact global cognitive performance, evaluated by MMSE scores. Our results suggest that hand dexterity, which was measured by the Purdue Pegboard Test, is vulnerable to the effects of aging and may be considered a measurable motor indicator for executive function impairment even in high cognitive functioning older adults. The findings provide a reasonable basis for implementing hand dexterity interventions for the prevention of executive function impairment in community-dwelling older adults.

## Abbreviations

ADLs: Activities of daily living
IADLs: Instrumental activities of daily living
MMSE: Mini-Mental State Examination
PPT: Purdue Pegboard Test
TMIG-IC: Tokyo Metropolitan Institute of Gerontology-Index of Competence
TMT-A: Trail Making Test-Part A
TMT-B: Trail Making Test-Part B
WAIS-R: Wechsler Adult Intelligence Test-Revised
