# Correction to: Hand dexterity, not handgrip strength, is associated with executive function in Japanese community-dwelling older adults: a cross-sectional study

**DOI:** 10.1186/s12877-018-0907-z

**Published:** 2018-09-11

**Authors:** Kimi Estela Kobayashi-Cuya, Ryota Sakurai, Naoko Sakuma, Hiroyuki Suzuki, Masashi Yasunaga, Susumu Ogawa, Toru Takebayashi, Yoshinori Fujiwara

**Affiliations:** 1 Research Team for Social Participation and Community Health, Tokyo Metropolitan Institute of Gerontology, 35-2 Sakae-cho, Itabashi-ku, Tokyo, 173-0015 Japan; 2 Department of Preventive Medicine and Public Health, School of Medicine, Keio University, 35 Shinanomachi, Shinjuku-ku, Tokyo, 160-8582 Japan

## Correction

Following the publication of this article [1], the authors reported a typesetting error in Table 1 that resulted in missing asterisks. This correction shows both the incorrect and correct versions of Table 1, and Table 1 has also been corrected in the original article.

The incorrect version is:

**Table 1:** Comparison of covariates, hand and cognitive variables among age categories (*N* = 326)

Variables	Age categories				*p*-value
	60-64 (*n* = 54)	65-69 (*n* = 104)	70-74 (*n*= 101)	≥ 75 (*n* = 67)	
Years of education	14.4 ± 2.5	13.4 ± 2.2	13.3 ± 2.5	12.8 ± 2.7	0.004
TMIG-IC	12.2 ± 0.8	12.3 ± 0.9	12.4 ± 0.9	12.2 ± 1.3	0.645
GDS	2.3 ± 2.1	3.1 ± 2.5	2.5 ± 2.3	3.0 ± 2.3	0.09
Heart disease, *n* (%)	2 (3.7)	1 (1.0)	11 (10.9)	7 (10.4)	0.012^a^
Diabetes, *n* (%)	2 (3.7)	9 (8.7)	6 (5.9)	5 (7.5)	0.670^a^
Hypertension, *n* (%)	11 (20.4)	23 (22.1)	27 (26.7)	28 (41.8)	0.020^a^
Stroke, *n* (%)	3 (5.6)	6 (5.8)	5 (5.0)	3 (4.5)	0.983^a^
Hand motor variables, mean ± SD					
Handgrip strength (Kg)	23.4 ± 4.7	22.6 ± 5.3	22.9 ± 7.0	20.9 ± 6.3	0.104
PPT (Number of pegs)	14.5 ± 1.5	13.7 ± 2.1	13.1 ± 1.9	11.9 ± 1.9^†,‡^	<0.001
Cognitive variables, mean ± SD					
MMSE	29.2 ± 0.9	28.8 ±1.3	28.9 ± 1.0	28.6 ± 1.3	0.023
TMT-A	30.2 ± 7.7	35.3 ± 13.2	37.9 ± 11.1	43.2 ±12.4^†,‡^	<0.001
TMT-B	74.5 ± 18.6	91.9 ± 37.6	107.4 ± 39.9^†^	121.6 ±40.8^†^	<0.001
Digit symbol^b^	67.7 ± 9.3	60.2 ± 13.8	55.3 ± 10.9	45.6 ± 8.4^†,‡^	<0.001

Values are expressed as mean ± SD

*TMIG-IC* Tokyo Metropolitan Institute of Gerontology - Index of Competence, *GDS* Geriatric Depression Scale, *MMSE* Mini-Mental State Examination, *TMT* Trail Making Test

Bonferroni correction for post-hoc tests: *p<0.008 vs. 60-64; ^†^*p*<0.008 vs. 65-69; ^‡^*p*<0.008 vs. 70-74

^a^The Chi-square test was performed

^b^The total number of subjects analyzed was 207

The correct version is:

**Table 1:** Comparison of covariates, hand and cognitive variables among age categories (*N* = 326)

Variables	Age categories				*p*-value
	60-64 (*n* = 54)	65-69 (*n* = 104)	70-74 (*n* = 101)	≥ 75 (*n* = 67)	
Years of education	14.4 ± 2.5	13.4 ± 2.2	13.3 ± 2.5*	12.8 ± 2.7*	0.004
TMIG-IC	12.2 ± 0.8	12.3 ± 0.9	12.4 ± 0.9	12.2 ± 1.3	0.645
GDS	2.3 ± 2.1	3.1 ± 2.5	2.5 ± 2.3	3.0 ± 2.3	0.09
Heart disease, *n* (%)	2 (3.7)	1 (1.0)	11 (10.9)	7 (10.4)	0.012^a^
Diabetes, *n* (%)	2 (3.7)	9 (8.7)	6 (5.9)	5 (7.5)	0.670^a^
Hypertension, *n* (%)	11 (20.4)	23 (22.1)	27 (26.7)	28 (41.8)	0.020^a^
Stroke, *n* (%)	3 (5.6)	6 (5.8)	5 (5.0)	3 (4.5)	0.983^a^
Hand motor variables, mean ± SD					
Handgrip strength (Kg)	23.4 ± 4.7	22.6 ± 5.3	22.9 ± 7.0	20.9 ± 6.3	0.104
PPT (Number of pegs)	14.5 ± 1.5	13.7 ± 2.1	13.1 ± 1.9*	11.9 ± 1.9*^,†,‡^	<0.001
Cognitive variables, mean ± SD					
MMSE	29.2 ± 0.9	28.8 ±1.3	28.9 ± 1.0	28.6 ± 1.3*	0.023
TMT-A	30.2 ± 7.7	35.3 ± 13.2	37.9 ± 11.1*	43.2 ±12.4*^,†,‡^	<0.001
TMT-B	74.5 ± 18.6	91.9 ± 37.6*	107.4 ± 39.9*^,†^	121.6 ±40.8*^,†^	<0.001
Digit symbol^b^	67.7 ± 9.3	60.2 ± 13.8	55.3 ± 10.9*	45.6 ± 8.4*^,†,‡^	<0.001

Values are expressed as mean ± SD

*TMIG-IC* Tokyo Metropolitan Institute of Gerontology - Index of Competence, *GDS* Geriatric Depression Scale, *MMSE* Mini-Mental State Examination, *TMT* Trail Making Test

Bonferroni correction for post-hoc tests: *p<0.008 vs. 60-64; ^†^*p*<0.008 vs. 65-69; ^‡^*p*<0.008 vs. 70-74

^a^The Chi-square test was performed

^b^The total number of subjects analyzed was 207
